# Supplementary material for: A qualitative evaluation of a compassion-focused therapy group intervention for UK healthcare staff at an acute hospital trust
Source: PLoS One. 2025 Sep 29;20(9):e0333582. doi: 10.1371/journal.pone.0333582 (PMC12478920; doi:10.1371/journal.pone.0333582)
Supplement: S1 File — (DOCX) [file pone.0333582.s001.docx]

**S1 File. Interview schedule**

| **MAIN QUESTIONS** | **PROBES AND PROMPTS** |
| --- | --- |
| To begin with when did you attend the groups? | |
| General feedback | |
| Starting quite broad, what was your overall experience of taking part in the CFT group? | How so? |
| Content  The next few questions will focus on the content and topic covered in the group. | |
| In terms of the content and topics covered in the groups…  Is there any part of the content or the topics covered that were particularly helpful? | - How so? How was it helpful?  - Could you tell me a bit more? |
| In the group, there was an emphasis on how it is to be compassionate to ourselves, and how we might experience individual blocks, fears and resistances to compassion | |
| How was your experience learning about these?  - How did you feel learning about compassion towards oneself?  - How did you feel learning about blocks/fears/resistances to compassion? | - Was there anything that stood out for you?  - Could you tell me a bit more? |
| In the group, there was also an emphasis on self-criticism and shame. | |
| How was your experience learning about self-criticism and shame? | - How did you feel learning about ….?  - Was there anything that stood out for you?  - Could you tell me a bit more? |
| In the group, there was also a discussion about the different parts, or selves, that exist within us and how they might think, feel, and respond in a situation. | |
| How did you find the focus on understanding the “critical self”?  What does a “critical self” might look like *for you?* | - How did you understand this concept?  - Did it resonate with you? How?  -Was there something challenging about this? |
| Do you feel you were able to build up a “compassionate self” over the course of the group?  What does a “compassionate self” might look like for *you*?  Have you been able to use this in life outside of the group? | -Could you tell me a bit more?  -Could you give me an example? |
| We talked about different topics covered in the group.  Is there any part of the topics covered that you think should be changed for future groups? |  |
| Group Format  Moving on, I am interested in knowing more about how it was for you to learn about all these topics as part of a group. | |
| What was it like listening to others’ experiences and sharing your own experiences in a group setting?   1. How was your experience being part of a group, learning together about compassion? 2. How was your experience being part of a group, learning together about self-criticism and shame?   Was there anything helpful about being in a group?  Was there anything unhelpful about being in a group? | - How so? In what way it was X? |
| Perceived changes | |
| Have you noticed any changes in your well-being as a result of attending this group? | - What have you noticed?  - Could you give me an example? |
| Practices | |
| Were there any particular practices or exercises that you learnt from attending the group that you have incorporated into your life?  *Follow-up questions*:  (a) What were the most helpful exercises from attending the group?  (b) What were the least helpful exercises from attending the group? | - How was X helpful?  - Could you give an example? |
| Suggested changes | |
| Is there anything that could have been done differently to improve the experience of being in a group? | - What? How? |
| Do you have any suggestions about how to make the group more useful for staff who attend in the future? |  |
| Take-away message | |
| Do you have a takeaway message or experience from being part of the group?  If yes, what is that?  Is there anything else that you think might be helpful for us to think about? Have I missed anything that feels important to mention? | |
