# Supplementary material for: A qualitative evaluation of a compassion-focused therapy group intervention for UK healthcare staff at an acute hospital trust
Source: PLoS One. 2025 Sep 29;20(9):e0333582. doi: 10.1371/journal.pone.0333582 (PMC12478920; doi:10.1371/journal.pone.0333582)
Supplement: S2 Table — (DOCX) [file pone.0333582.s003.docx]

**S3 Table. Thematic analysis table presenting number of participants mentioning theme and subtheme by research question**

| **Research questions** | **Themes** | **Subthemes** | **Number of participants [ID]** | **Example quote** |
| --- | --- | --- | --- | --- |
| What was the experience of staff who took part in the CFT group? | 1. **Group acceptability** | - 1. Developing group safeness | 8 | “*I had somewhere to be authentically me and safe*” (Participant 7) |
|  |  | - 1. Affiliative experience | 8 | “*It did feel like being in a bit of a family in that way as time went on. To begin with, it felt like a disparate group of people, but the time went on and it felt like a group of NHS people who would all have stuff that meant they were needing a bit of support*.” (Participant 4) |
|  |  | - 1. Unexpected challenges | 4 [1,3,6,7] | *“I wasn't expecting to bring up so many bad feelings and maybe it should come with a warning as such [laughing]. Because I know other people did experience that, that it really triggered some -although I suppose you to get over it, you gotta address it.”* (Participant 1) |
| How did staff experience learning about different concepts of CFT? | 1. **Usefulness of engaging with key CFT content** | - 1. Helped make sense of personal experiences | 8 | *“it's definitely started the work on self-compassion. Particularly understanding how brains work and that kind of like, rather than it being my fault, it's, this is what you're what your brain, this is what brains do when they've been through very difficult situations, so less work on me as an individual, but more work on understanding that that this is what happens and beginning to develop tools”* (Participant 4) |
|  |  | - 1. Building a helpful definition self-compassion | 5 [2,3,4,6,7] | “*I think my biggest take away, because I always felt that compassion or feel that compassion was pink, fluffy and roses and hearts and loves and kisses and that sort of thing which you know to some extent is but also the compassion can be strong and standing up for yourself. And that was like, wow, never even thought of it like that*” (Participant 3) |
|  |  | - 1. Increasing self-awareness | 7 [1,2,3,4,5, 6,7] | “[…] *that kind of compassion was re-, like, focusing on yourself and giving yourself compassion was really, really hard, but realising how little I had of it towards me, I think*” (Participant 5) |
|  |  | - 1. Offered ways to manage difficult inner experiences | 7 [2,3,4,5,6,7, 8] | *“So knowing that I could effectively separate, that's that really defensive, self-critical side to my compassionate side and have them talk in like a really open way was really helpful to be actually “Yes. It's a part of me, but it's not that big a part of me. It's how much I want it to be me”, if that makes sense. So that was a really powerful lesson I think.”* (Participant 7) |
|  | 1. **Challenges engaging with particular CFT content** |  | 6 [2,3,4,5,6, 8] | “*That would be the one that opened the can of worms was to take me back to a place where I thought that I could remember when the self-criticism started. That was by far the hardest week, by far the hardest activity. But, on the flip side of that, it has, then had that rollercoaster of getting support to unpack that can of worms*.” (Participant 6) |
| How did staff experience working on CFT concepts as part of a group? | 1. **Universality** |  | 8 | “*I think what I found most helpful about that group is that I realised that lots of people struggle with similar things to me, and actually that inner critic in everybody is quite prevalent. [And it was helpful] to be seen, isn't it? It's to be seen and not to be alone, I think.”* (Participant 8) |
|  | 1. **Learning with and from each other** |  | 8 | *“I do remember having a few light bulb moments when they’d say something you think “ohh yeah. Oh yeah”, you know? […] So, you were able to bounce things off, that you, that you didn't wouldn't, wouldn't have thought about that. Somebody else says something triggered something and you thought, ”Oh yeah, that makes sense now”.”* (Participant 1) |
| If any, what was the impact of being part of the group on their well-being? | 1. **Cultivated compassion** | - 1. Recognising the need for self-compassion | 4 [1,2,3,5] | *“[…] difficult, but definitely necessary to face the fact that we need to take care of ourselves too. To take care of myself too and have compassion about me and my thoughts and what I'm going through*.” (Participant 2) |
|  |  | - 1. Feeling deserving of self-compassion | 8 | “*I think I've put into place some things. I no longer feel selfish. I think it was separating out. […]. It's giving me permission to. Go off and be compassionate to myself, knowing that that's actually really good and healthy and OK, so yeah, so I probably have said no to things without then having guilt from saying no or thinking, oh, should I have done that? You know all those sorts of things? It's a no*.” (Participant 3) |
|  |  | - 1. Responding with self-compassion | 7 [1, 2,3,4,5,6,7] | *“I do feel kinder to myself. I don't feel I put as much pressure on myself. Definitely if I do things wrong, I just think, “OK, fine”. You know, “it’s a new day tomorrow” and I do feel like that. Uhm, I wouldn't say it's easy, but I am getting better at it, yeah*.” (Participant 1) |
|  | 1. **Acquired skills to support well-being** |  | 8 | “*I've connected with the breathing and stuff just as the- It's not quick and easy, but like, very physical way of connecting with the compassion itself and just kind of letting go of the things that aren't as important during my day*.” (Participant 7) |
|  | 1. **Emotional improvement** |  | 4 [2,4,6,7] | “*I went through a bit of a dark place, and I really struggled at times, it also helped in a sense, because I was able to take, like I said, some of the strategies […] So although it felt like a bit of a dark place actually it helped me through quite a dark place*.” (Participant 6) |
|  | 1. **Group as the start of an ongoing therapeutic journey** |  | 5 [2,4,5,6,8] | “*It started me on the journey I would say. I think if I hadn't gone to that group, I don't think, I probably would have just thought right just get on with it, um, but I think going to that group made me realise things weren't OK. Umm. And it was almost OK to get help and start to think about these things*” (Participant 5) |
| What are participants' views on improving the experience of CFT groups in the future? | 1. **Future delivery** | - 1. Adapting content | 6 [1,2,3,4,7,8] | “*It was really good. And that was because of the people who attended. And because of [clinician’s name], but I think. Only because [clinician’s name] allowed the people within the group to shape the discussion so much, and I think that was really lovely*” (Participant 8) |
|  |  | - 1. Further support | 7 [1,2,3,4,5,6,7] | “*A place to remind you of the things that you've learnt but you know, remind you again of the self-critical that might have tripped back in and things like that. Almost like little top ups*.” (Participant 3) |
|  |  | - 1. Supporting group safeness | 6 [2,3,4,5,6,7] | “*it's very difficult to me and I think for the other people that was there to allocate time to take care of ourselves and to address those kind of needs. And we had that protected time for those, was it 8 weeks? I can't remember very well. But for that time that the course happened, we had that protected time that everyone knew that we needed to go there.”* (Participant 2) |
|  |  | - 1. Group set-up | 7 [1,2,3,5,6,7,8] | “*The smaller group did feel more intimate, and I feel people felt that barriers were broken down. You could be a little bit more you with a smaller group of people*.” (Participant 1) |
| *Note.* ID = Participant identification number | | | | |
